# Mechanisms of dual modulatory effects of spermine on the mitochondrial calcium uniporter complex

**DOI:** 10.1016/j.jbc.2025.108218

**Published:** 2025-01-23

**Authors:** Yung-Chi Tu, I-Chi Lee, Tsai-Wei Chang, Vivian Lee, Fan-Yi Chao, Eitel R. Geltser, Ming-Feng Tsai

**Affiliations:** 1 Department of Physiology and Biophysics, University of Colorado Anschutz Medical Campus, Aurora, Colorado, USA; 2 Department of Molecular Physiology and Biological Physics, University of Virginia School of Medicine, Charlottesville, Virginia, USA; 3 School of Medicine, National Yang Ming Chiao Tung University, Taipei, Taiwan

**Keywords:** calcium channel, mitochondrial transport, polyamine, membrane biophysics, channel activation, cell signaling

## Abstract

The mitochondrial Ca^2+^ uniporter is the Ca^2+^ channel responsible for mitochondrial Ca^2+^ uptake. It plays crucial physiological roles in regulating oxidative phosphorylation, intracellular Ca^2+^ signaling, and cell death. The uniporter contains the pore-forming MCU subunit, the auxiliary EMRE protein, and the regulatory MICU1 subunit, which blocks the MCU pore under resting cellular Ca^2+^ concentrations. It has been known for decades that spermine, a biological polyamine ubiquitously present in animal cells, can enhance mitochondrial Ca^2+^ uptake, but the underlying mechanisms remain incompletely understood. In this study, we demonstrate that spermine exerts both potentiation and inhibitory effects on the uniporter. At physiological concentrations, spermine binds to membranes and disrupts MCU–MICU1 interactions, thereby opening the uniporter to import more Ca^2+^. However, at millimolar concentrations, spermine also inhibits the uniporter by targeting the pore-forming region in a MICU1-independent manner. These findings provide molecular insights into how cells can use spermine to control the critical processes of mitochondrial Ca^2+^ signaling and homeostasis.

The mitochondrial calcium uniporter (hereafter referred to as the uniporter) is a multisubunit Ca^2+^ channel that mediates mitochondrial Ca^2+^ uptake across the inner mitochondrial membrane (IMM). Upon receiving intracellular Ca^2+^ signals, the uniporter becomes activated to rapidly transport Ca^2+^ into the mitochondrial matrix, thereby modulating the magnitude and frequency of these Ca^2+^ signals (1, 2). This influx of Ca^2+^ can also elevate matrix Ca^2+^ levels to stimulate Ca^2+^-regulated dehydrogenases in the TCA cycle and enhance oxidative phosphorylation (1, 2). However, excessive Ca^2+^ entry can trigger the mitochondrial permeability transition, leading to apoptotic cell death (1, 2). Dysfunction of the uniporter has been implicated in a range of pathologies (3, 4), including cardiac ischemia-reperfusion injury, heart failure, neurodegeneration, and cancer metastasis, among others. Given the uniporter’s critical importance in physiology and disease, it is crucial to understand the mechanisms by which the uniporter is regulated.

It has been known for decades that the uniporter is regulated by cytoplasmic Ca^2+^. Following the identification of uniporter genes in the early 2010s (5, 6, 7, 8), extensive research has established an “occlusion” mechanism underlying such Ca^2+^ regulation (9, 10, 11, 12, 13, 14, 15, 16, 17, 18, 19, 20). As illustrated in Fig. 1*A*, the uniporter remains quiescent at resting cellular Ca^2+^ concentrations ([Ca^2+^]) of ∼100 nM. This inactivity is due to the MICU1 subunit, which can form a homodimer or heterodimerize with MICU2 in the intermembrane space (IMS), obstructing the IMS entrance of the uniporter’s Ca^2+^ pore formed by the MCU subunit. When cytoplasmic Ca^2+^ signals elevate IMS [Ca^2+^], Ca^2+^ binding to MICU1 causes MICU1 separation from MCU, thus leading to opening of the pore and activation of the uniporter. In this Ca^2+^-activated state, MICU1 remains bound to the EMRE subunit through electrostatic interactions, ensuring continued MICU1 association within the uniporter complex. Consequently, once the Ca^2+^ signal is over, MICU1 can rapidly block the pore to close the uniporter.

**Figure 1:**
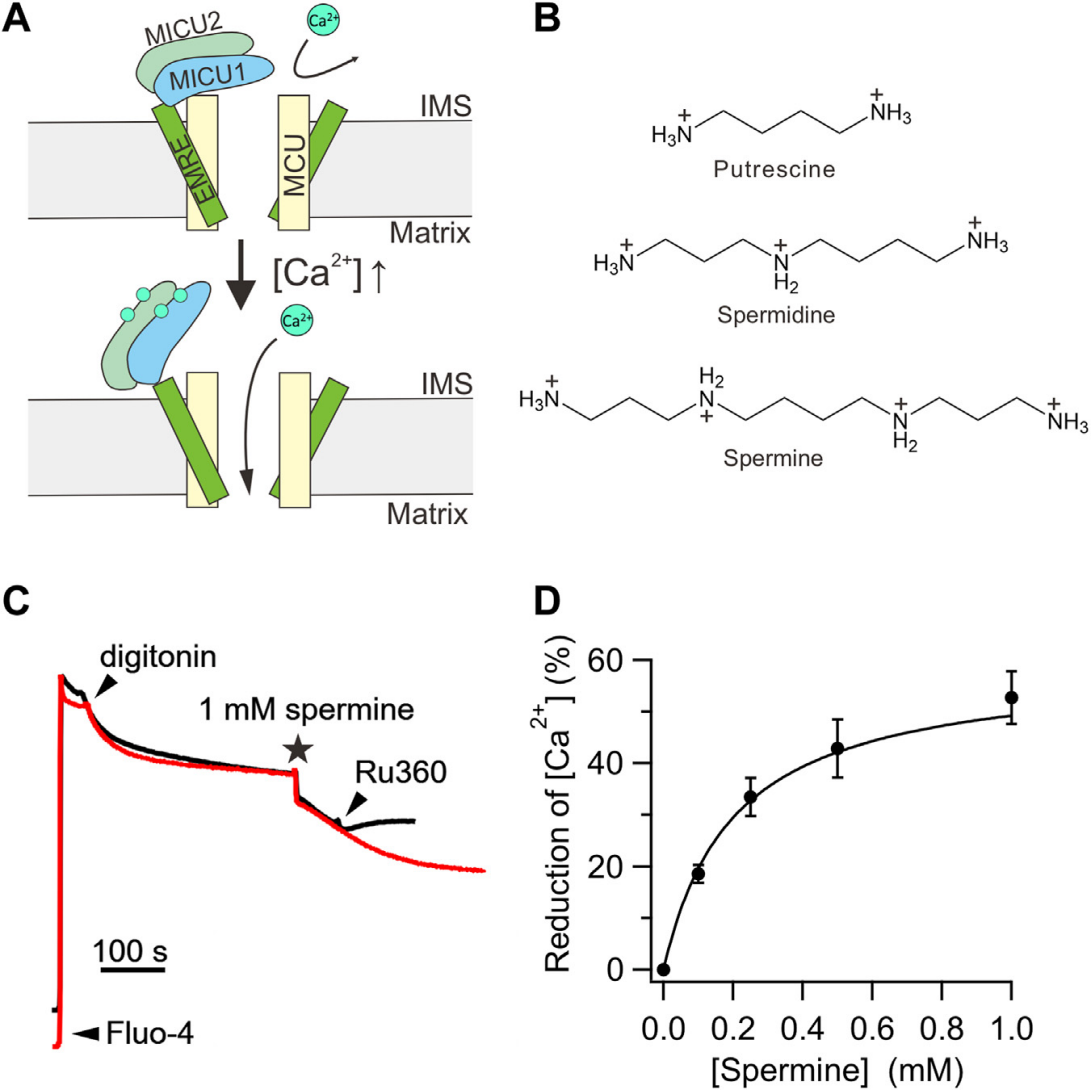
**Regulation of mitochondrial calcium uptake by Ca**^**2+**^**or spermine.***A*, molecular model depicting Ca^2+^-dependent activation of the uniporter. At resting cellular Ca^2^⁺ levels (*top*), the channel is blocked by its MICU1 subunit. When intracellular Ca^2^⁺ signals elevate the local [Ca^2^⁺] around the uniporter, the MICU1 block is relieved, allowing the channel to open (*bottom*). *B*, chemical structures of common biological polyamines. *C*, spermine enhancement of mitochondrial Ca^2+^ buffering. The addition of 1 mM spermine to digitonin-permeabilized WT cells causes mitochondria to sequester more Ca^2+^. This effect is abolished by 100 nM Ru360, as shown by the *black trace*. The y-axis is presented in arbitrary units (A.U.). *D*, dose–response relationship for spermine enhancement of mitochondrial Ca^2+^ buffering. The graph illustrates the correlation between spermine concentration (X) and the percentage reduction in [Ca^2+^]_ex_ (Y) induced by spermine. Data fitting (*black curve*) was done using a single-site binding equation: Y(X) = Y_max_・X/(X + EC_50_), where Y_max_ is the maximal percentage reduction and EC_50_ is the half-maximal effective concentration. Data are presented as mean ± standard deviation (S.D.), with each data point representing a minimum of three independent biological replicates. EC_50,_ half-maximal effective concentration; IMS, intermembrane space.

Here, we investigate a possible uniporter regulator, spermine, which, along with spermidine and putrescine (Fig. 1*B*), are important biological polyamines ubiquitously present in animal cells, playing critical roles in cell growth, differentiation, protein synthesis, and apoptosis (21, 22, 23, 24). As a positively charged molecule, spermine is known to modulate the activity of various cation channels by blocking their pores. For example, it imparts inward-rectification properties to multiple inward-rectifier K^+^ (K_ir_) channels and AMPA-/kainate-type glutamate receptor channels by entering the pore from the intracellular side to block outward currents during membrane depolarization (25, 26, 27, 28, 29, 30). Spermine block of cyclic nucleotide-gated channels (31), voltage-gated Na^+^ channels (32, 33), and transient receptor potential channels (34, 35) has also been described before.

Interestingly, Nicchitta and Williamson (36) reported in 1984 that spermine can enhance the ability of mitochondria to buffer extramitochondrial Ca^2+^. Their study showed that isolated liver mitochondria, or mitochondria in permeabilized hepatocytes, could buffer extramitochondrial [Ca^2+^] ([Ca^2+^]_ex_) to a steady-state level of 0.5 to 1 μM. However, the addition of spermine allows mitochondria to sequester more Ca^2+^, further lowering [Ca^2+^]_ex_ to 0.2 to 0.5 μM. They also found that spermidine exhibited ∼5-fold lower efficacy and potency compared to spermine, whereas putrescine was ineffective. These initial observations have since been verified across multiple laboratories (37, 38, 39, 40, 41, 42, 43).

The ability of mitochondria to buffer external Ca^2+^ is governed by the balance between uniporter-mediated mitochondrial Ca^2+^ uptake and Ca^2+^ efflux through IMM Ca^2+^ transporters (44). While mitochondrial Ca^2+^ efflux appears unaffected by spermine (36), kinetic analyses of how spermine impacts mitochondrial Ca^2+^ uptake have yielded contradictory results. Some studies found that spermine increases the rate of mitochondrial Ca^2+^ uptake (40), while others reported only inhibitory effects (37, 38, 45). Moreover, some researchers observed more complicated phenomena, with spermine increasing the rate of mitochondrial Ca^2+^ uptake when [Ca^2+^]_ex_ is below 5 to 10 μM but reducing it at higher [Ca^2+^]_ex_ (36, 41, 42). Research in this area largely ceased in the 1990s, leaving key mechanistic questions unanswered. First, does spermine actually regulate the uniporter, and if so, what are the underlying molecular mechanisms? Moreover, how can we reconcile the conflicting data on spermine’s influence on mitochondrial Ca^2+^ uptake kinetics?

We sought to address these questions for two reasons. First, from a mechanistic standpoint, the possibility that spermine may enhance uniporter function—rather than simply blocking the pore as it does in other cation channels—offers an intriguing direction for investigation in channel biophysics. Second, the regulation of mitochondrial Ca^2+^ buffering by spermine is physiologically relevant, as its half-maximal effective concentration (EC_50_) of 100 to 200 μM aligns with the range of free spermine concentrations in the cytoplasm (46). Therefore, gaining a deeper understanding of spermine potentiation could significantly advance our knowledge of how cells regulate the vital processes of cytoplasmic and mitochondrial Ca^2+^ signaling and homeostasis.

## Results

### Spermine perturbs MICU1 block of the uniporter

To examine spermine potentiation of mitochondrial Ca^2+^ buffering, we permeabilized the plasma membrane of wildtype (WT) human embryonic kidney 293 cells with digitonin to expose mitochondria to extracellular Ca^2+^ and used Fluo-4 to monitor changes in [Ca^2+^]_ex_. Fig. 1*C* shows that mitochondria reduced [Ca^2+^]_ex_ to a steady-state level (517 ± 27 nM), and subsequent addition of 1 mM spermine further decreased [Ca^2+^]_ex_ by ∼50% (255 ± 8 nM) (see Experimental Procedures for calculation details). Dose–response experiments relating spermine concentrations to [Ca^2+^]_ex_ reduction yielded an EC_50_ of 201 μM (Fig. 1*D*). These results are in good agreement with the original data from Nicchitta and Williamson (36). We further show that the stimulatory effects of spermine depend on the uniporter, as applying the uniporter inhibitor azanide;formic acid;ruthenium(5+);trichloride;hydrate (Ru360) prevents spermine from enhancing mitochondrial Ca^2+^ sequestration (black trace, Fig. 1*C*).

**Figure 2:**
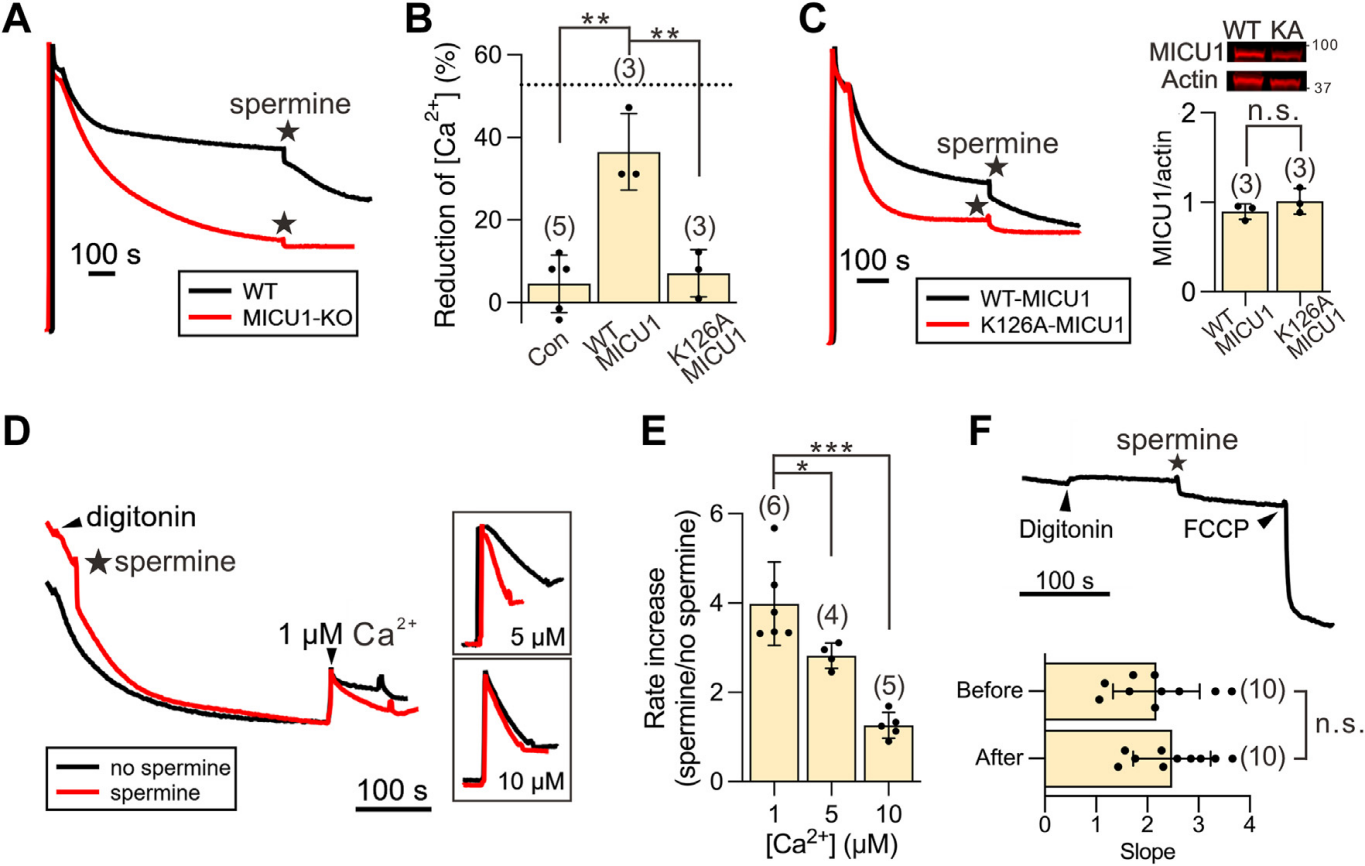
**Spermine perturbation of MICU1-mediated block of the uniporter.***A*, the requirement of MICU1 for spermine potentiation effects. The addition of 1 mM spermine (indicated by a *star*) does not enhance mitochondrial Ca^2^⁺ uptake in MICU1-KO cells. *B*, bar chart summarizing the ability of spermine to reduce [Ca^2+^]_ex_. *Left*: MICU1-KO cells. *Middle*: MICU1-KO cells transfected with WT MICU1. *Right*: MICU1-KO cells transfected with K126A MICU1. *Dashed line*: WT HEK cells as in Fig. 1, *C* and *D*. *C*, impact of the MICU1 K126A mutation on spermine’s stimulatory effect. Representative traces show spermine-induced changes in [Ca^2^⁺]ₑₓ for MICU1-KO cells transfected with WT or K126A MICU1. Both MICU1 constructs were C-terminally FLAG tagged and expressed at similar levels as detected by Western blot using anti-FLAG antibody. KA: K126A. Bar chart shows quantification of MICU1 signals normalized to actin (loading control). *D*, spermine-induced acceleration of mitochondrial Ca^2+^ uptake. Various concentrations of Ca^2+^ were added to induce net mitochondrial Ca^2+^ uptake in the presence (*red*) or absence (*black*) or spermine. Inlets highlight the traces following the addition of 5 or 10 μM Ca^2+^. *E*, fold increase in mitochondrial Ca^2+^ uptake rate in response to spermine. The bar chart quantifies the results from the experiments shown in Panel *D*. Fold increases were determined by calculating the ratio of the mitochondrial uptake rate in the presence of spermine to that in its absence. *F*, lack of spermine effects on IMM potentials. TMRM-loaded cells were permeabilized with digitonin and treated with spermine. The bar chart shows the slopes before and after spermine addition, indicating no significant difference in IMM potentials. The H⁺-ionophore FCCP was added at the end of the experiment to fully collapse the membrane potential. 1 mM spermine was used throughout the figure. The y-axes in panels *A*, *C*, *D*, and *F* are in A.U. Statistical analysis was performed using two-tailed, unpaired *t* test, with significance levels denoted as: ∗*p* < 0.05, ∗∗*p* < 0.01, ∗∗∗*p* < 0.001, and n.s. (not significant). Individual data points, representing independent biological replicates, are superimposed on the bar charts. FCCP, carbonyl cyanide-4-(trifluoromethoxy)phenylhydrazone; TMRM, tetramethylrhodamine, methyl ester; IMM, inner mitochondrial membrane.

It has been known that MICU1 knockout (KO) allows mitochondria to buffer [Ca^2+^]_ex_ to lower levels (Fig. 2*A*) by preventing occlusion of the uniporter by MICU1 as [Ca^2+^]_ex_ decreases (Fig. 1*A*). The striking similarity between the effects of spermine addition and MICU1 KO led us to hypothesize that spermine might act by disrupting MICU1 block of the MCU pore. This hypothesis predicts that spermine would have no effect on mitochondrial Ca^2+^ absorption when the uniporter is not blocked by MICU1, specifically (1) in MICU1-KO conditions, (2) when WT MICU1 is replaced by a K126A MICU1 mutant incapable of blocking MCU(12), or (3) at elevated [Ca^2+^]_ex_, which separates MICU1 from the MCU pore.

Our experiments strongly support this hypothesis. First, we observed that mitochondria lose responses to spermine in the absence of MICU1 (Fig. 2, *A* and *B*) and that this phenotype can be rescued by expressing WT MICU1 but not the K126A mutant (Fig. 2, *B* and *C*). Moreover, the stimulation of mitochondrial Ca^2+^ uptake by spermine decreased progressively as [Ca^2+^]_ex_ increased from 1 μM to 5 μM, and then to 10 μM (Fig. 2, *D* and *E*). Collectively, these results demonstrate that spermine enhances mitochondrial Ca^2+^ buffering by disrupting MICU1 block of the uniporter. Corroborating previous reports (36, 43), we found that spermine has no impact on the IMM potential (Fig. 2*F*), indicating that the observed spermine effects are not caused by altered driving force for Ca^2+^ influx.

### The mechanisms underlying spermine potentiation

**Figure 3:**
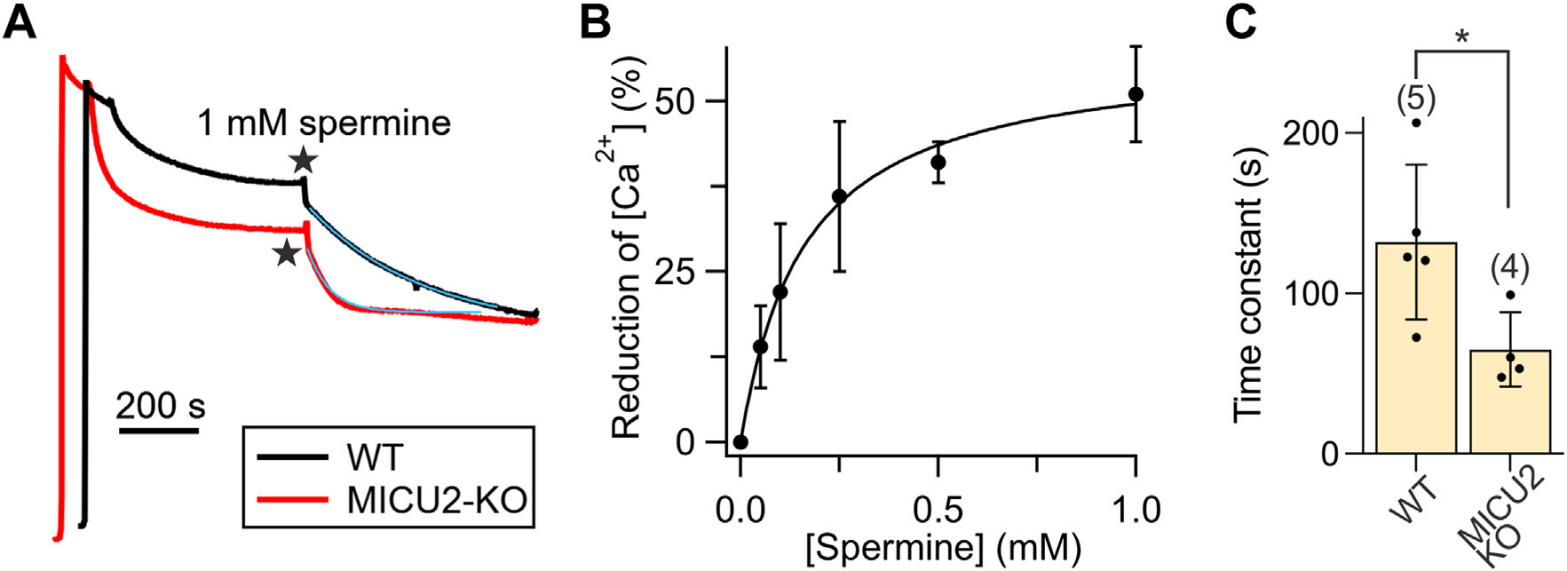
**Effects and kinetics of spermine in MICU2-KO cells.***A*, the effect of MICU2 KO on spermine potentiation. Representative traces illustrates the effects of 1 mM spermine (indicated by a *star*) on [Ca^2+^]_ex_ for digitonin-permeabilized WT and MICU2-KO cells. *Blue curves* represent single-exponential fits to the data. *B*, dose–response curve for spermine obtained using MICU2-KO cells. Data fitting (*black curve*) was performed as in Fig. 1*D*. *C*, time constants for spermine-induced [Ca^2+^]_ex_ reduction, derived from the single-exponential fits shown in panel A. Statistical analysis was conducted using two-tailed, unpaired *t* test with significance defined as ∗*p* < 0.05. Each dot on the bat chart represents an independent biological replicate.

Results described above have revealed that spermine potentiation requires MCU, EMRE, and MICU1 because the spermine effect can be eliminated by (1) Ru360 block of the uniporter pore, formed by MCU and EMRE (Fig. 1*C*) and (2) the removal of MICU1 (Fig. 2, *A* and *B*). In WT HEK cells, the uniporter is regulated by a MICU1-MICU2 heterodimer (MICU1-2) (Fig. 1*A*). To determine if MICU2 is necessary for spermine’s effects, we tested spermine on MICU2-KO HEK cells, where the uniporter contains a MICU1 homodimer (MICU1-1) (9, 47). In these cells, mitochondria reduced [Ca^2+^]_ex_ to 394 ± 43 nM, and the addition of 1 mM spermine further reduced [Ca^2+^]_ex_ to 211 ± 15 nM (Fig. 3*A*). The EC_50_ for spermine is 158 μM (Fig. 3*B*), comparable to that obtained in WT cells (Fig. 1*D*). These results indicate that MICU2 is not essential for spermine potentiation. Interestingly, the time course of spermine-induced [Ca^2+^]_ex_ reduction (blue curves in Fig. 3*A*) is ∼2-fold faster in MICU2-KO cells than in WT cells (Fig. 3*C*). This observation aligns with previous studies (9, 19) showing that MICU1-1 dissociates from MCU more readily than MICU1-2, suggesting that spermine may disrupt the occlusion of MCU by MICU1-1 more easily than by MICU1-2.

**Figure 4:**
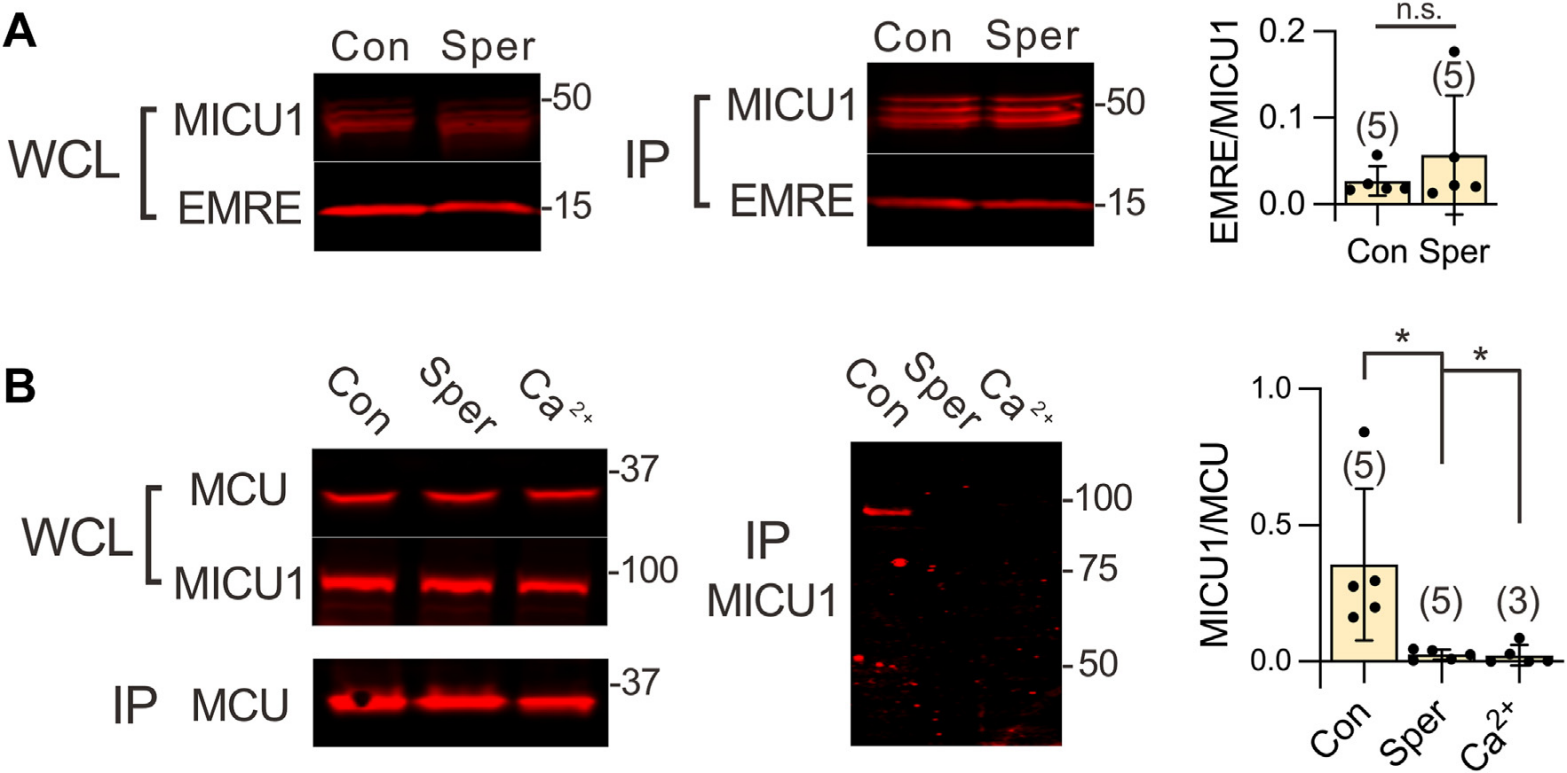
**Western blot analysis of spermine’s impacts on uniporter subunit interactions.***A*, spermine effects on MICU1–EMRE interactions. The indicated uniporter subunits were expressed in MICU1-MCU-EMRE-KO cells. MICU1 was used to pull down EMRE. A C463S MICU1 mutant, which cannot form a disulfide-connected MICU1-1 dimer, was used in this experiment. Similar results were obtained using WT MICU1. The bar chart quantifies the Western blot signal ratio of EMRE to MICU1 in the IP samples. *B*, disruption of the MCU–MICU1 complex by spermine. MCU was immobilized to pull down WT MICU1, co-expressed in MICU1-MCU-EMRE-KO cells. The MICU1 bands migrate at ∼100 kDa, representing a disulfide-linked MICU1-1 dimer. Con: 1 mM EGTA. Sper: 1 mM EGTA and 1 mM spermine. Ca^2+^: no EGTA and 10 μM added Ca^2+^. The MICU1-to-MCU ratio in the IP samples is quantified in the accompanying bar chart. MCU and EMRE were tagged with a C-terminal 1D4 sequence (TETSQVAPA), while MICU1 has a C-terminal FLAG tag. Detection was performed using antibodies against the respective tags. All experiments were performed with at least five independent repeats (additional data shown in Figs. S1 and S2). Statistical analysis was performed using a two-tailed paired *t* test, with significance defined as ∗*p* < 0.05. Individual dots in the bar charts represent independent biological replicates. IP, proteins obtained after CoIP; WCL, whole-cell lysate.

We explored two potential mechanisms by which spermine might disrupt MICU1 block of the uniporter. First, spermine could interfere with the EMRE-MICU1 interaction that anchors MICU1 within the uniporter complex, potentially leading to the formation of MICU1-free uniporters (15). Second, spermine might directly inhibit MICU1's binding to MCU. To investigate these possibilities, we conducted co-immunoprecipitation (CoIP) experiments to assess the impact of spermine on EMRE–MICU1 and MCU–MICU1 interactions. Results show that MICU1 binding to EMRE is insensitive to 1 mM spermine (Figs. 4*A* and S1). In contrast, spermine completely breaks the MCU–MICU1 complex, similar to how adding 10 μM Ca^2+^ dislodges MICU1 from MCU (Figs. 4*B* and S2). These findings led us to conclude that spermine enhances mitochondrial buffering of Ca^2+^ by perturbing the MCU–MICU1 interactions that otherwise shut the uniporter at low Ca^2+^.

**Figure 5:**
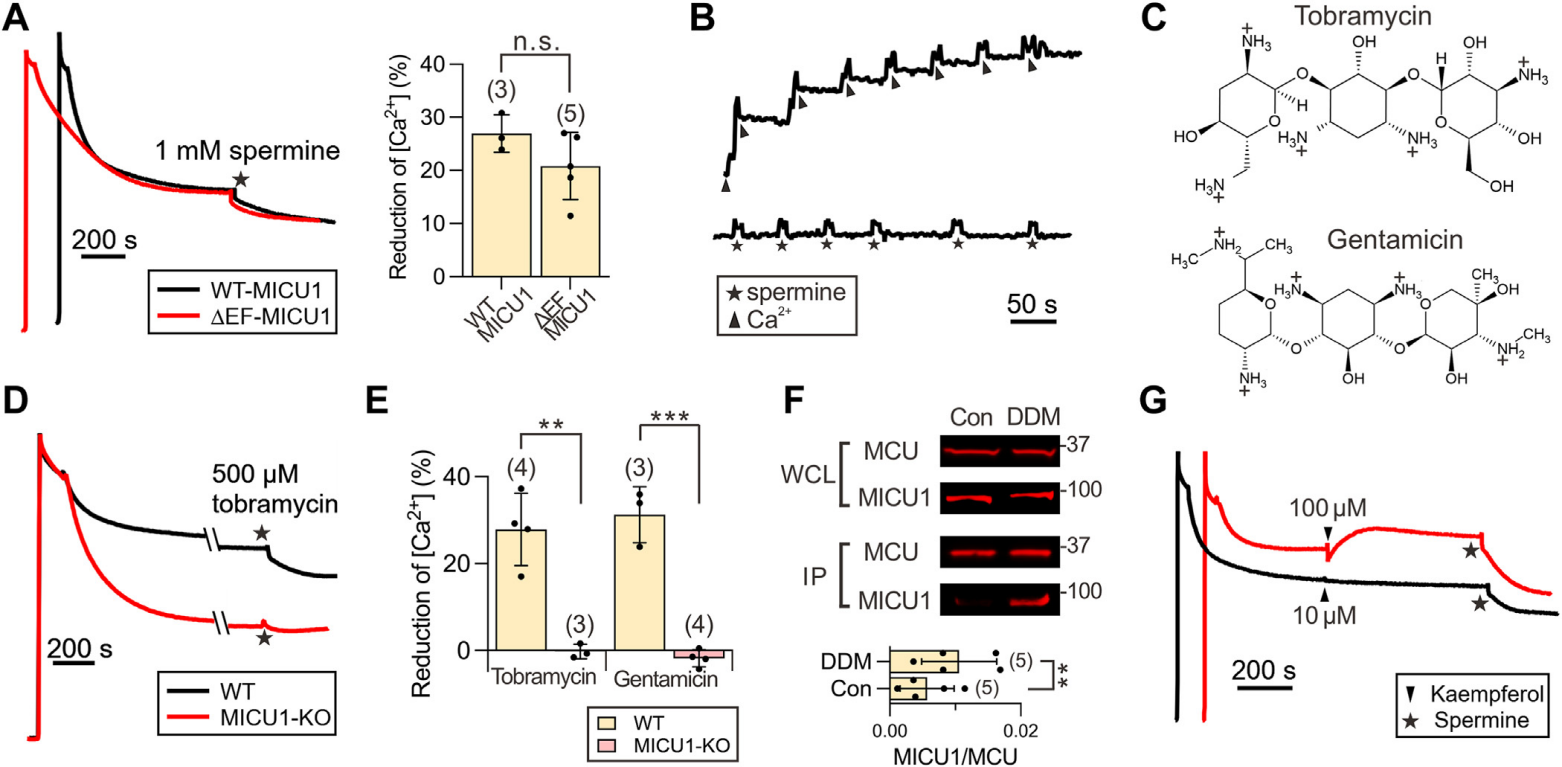
**Lipid dependence of spermine potentiation and analyses of other uniporter potentiators.***A*, roles of MICU1’s EF hands in spermine action. Representative traces show spermine potentiation in permeabilized MICU1-KO cells transiently expressing WT- or EF-hand disabled (ΔEF) MICU1. The accompanying bar chart reports spermine-induced reduction of [Ca^2+^]_ex_. *B*, changes in MICU1 tryptophan fluorescence upon addition of Ca^2+^ or spermine. Each addition represents 30 μM CaCl_2_ (*arrow heads*) or 0.5 mM spermine (*stars*). *C*, chemical structures of tobramycin and gentamicin. *D*, tobramycin potentiation of the uniporter abolished by MICU1-KO. *Stars* denote the addition of 0.5 mM tobramycin. *E*, bar chart summarizing the effects of tobramycin and gentamicin on [Ca^2+^]_ex_. 0.5 mM tobramycin and gentamicin were applied to permeabilized WT and MICU1-KO HEK cells. *F*, Western blot analyses showing essential roles of lipids in spermine-induced disruption of MCU-MICU1 interactions. Con: 1 mM spermine was added to digitonin-permeabilized cells for 10 min before solubilization with DDM. DDM: 1 mM spermine was added after digitonin-permeabilized cells were solubilized with DDM for 10 min. 1D4-tagged MCU in DDM solubilized samples was immobilized to pull down FLAG-tagged MICU1. Five independent experiments were performed, all yielding consistent results (additional data provided in Fig. S3). The bar chart summarizes the MICU1-to-MCU Western blot signal ratio in the IP samples. *G*, effects of kaempferol on mitochondria Ca^2+^ transport. Traces are representative of results from four independent experiments. Statistical analysis was performed using two-tailed, unpaired (panel *A* and *E*) or paired (panel *F*) *t* test. ∗∗*p* < 0.01; ∗∗∗*p* < 0.001; n.s., not significant. Each dot on the bar charts represents an independent biological replicate. DDM, n-dodecyl-β-D-maltopyranoside.

Where does spermine bind? Given spermine’s positive charge, we considered whether it might interact with the two EF-hand Ca^2+^-binding motifs in MICU1, potentially inducing a conformation resembling the Ca^2+^-bound state, which would be unable to block MCU. Accordingly, we tested spermine on a MICU1 mutant (ΔEF MICU1) with four mutations (D231A, E242K, D421A, and E423K) that disable both EF hands (18). As shown in Figure 1, Figure 5*A*, 1 mM spermine caused a similar reduction of [Ca^2+^]_ex_ in MICU1-KO cells expressing either WT or ΔEF MICU1. Additionally, while Ca^2+^ binding to the EF hands of purified MICU1 alters tryptophan fluorescence as reported previously (48), no such fluorescence change was observed with the addition of spermine (Fig. 5*B*), consistent with spermine’s effects not mediated through the EF hands. Taken together, these results demonstrate that spermine does not potentiate the uniporter by binding to MICU1’s EF hands or by modulating Ca^2+^ binding to these EF hands.

It has been shown that aminoglycoside antibiotics, such as tobramycin or gentamicin (Fig. 5*C*), can also improve mitochondrial Ca^2+^ buffering (37, 49). Our experiments not only confirm these findings (Fig. 5, *D* and *E*) but also reveal that the stimulatory effects of tobramycin and gentamicin are abolished in MICU1-KO cells (Fig. 5, *D* and *E*). These results suggest that aminoglycosides and spermine likely enhance mitochondrial Ca^2+^ buffering through a common mechanism involving the disruption of MICU1 block of the uniporter. Polyamines and aminoglycosides, while both highly charged, share no structural similarity (Fig. 1*B* *versus* Fig. 5*C*). Therefore, it is unlikely that MICU1 could contain a binding pocket that not only accommodates both types of compounds but also allows these compounds to produce similar effects.

In the literature, it has been firmly established that both spermine and aminoglycosides can interact with liposome or mitochondrial membranes (37, 40, 50, 51, 52, 53), raising the possibility that these compounds might bind to the IMM to disrupt MICU1 interactions with MCU. To test this, we performed CoIP under two conditions to assess spermine effects on the MCU–MICU1 complex. First, spermine was added to digitonin-permeabilized cells to bind the IMM before the samples were solubilized with n-dodecyl-β-D-maltopyranoside (DDM). Alternatively, spermine was added after DDM had solubilized all membranes in digitonin-permeabilized cells. Results show that spermine is more effective at breaking the MCU–MICU1 complex when the IMM remains intact (Figs. 5*F* and S3), consistent with lipid bilayers playing critical roles in spermine potentiation.

Some plant flavonoids, such as kaempferol, have been reported to stimulate the uniporter (54). These compounds are not charged and are not known to bind lipids. We thus predict that they would not enhance uniporter activity *via* mechanisms similar to spermine or aminoglycosides. Accordingly, we examined the effect of kaempferol on mitochondrial Ca^2+^ uptake. Fig. 5*G* shows that after mitochondria buffered [Ca^2+^]_ex_ to a steady state in permeabilized WT cells, adding 10 μM kaempferol fails to alter steady-state [Ca^2+^]_ex_. Interestingly, 100 μM kaempferol induces a slow Ca^2+^ efflux, suggesting that kaempferol might modulate Ca^2+^-efflux transporters. Adding spermine after kaempferol still causes a reduction of [Ca^2+^]_ex_, indicating that spermine and kaempferol act independently. Altogether, these results demonstrate that kaempferol, as anticipated, does not stimulate the uniporter in the same manner as spermine.

### Spermine also blocks the uniporter

**Figure 6:**
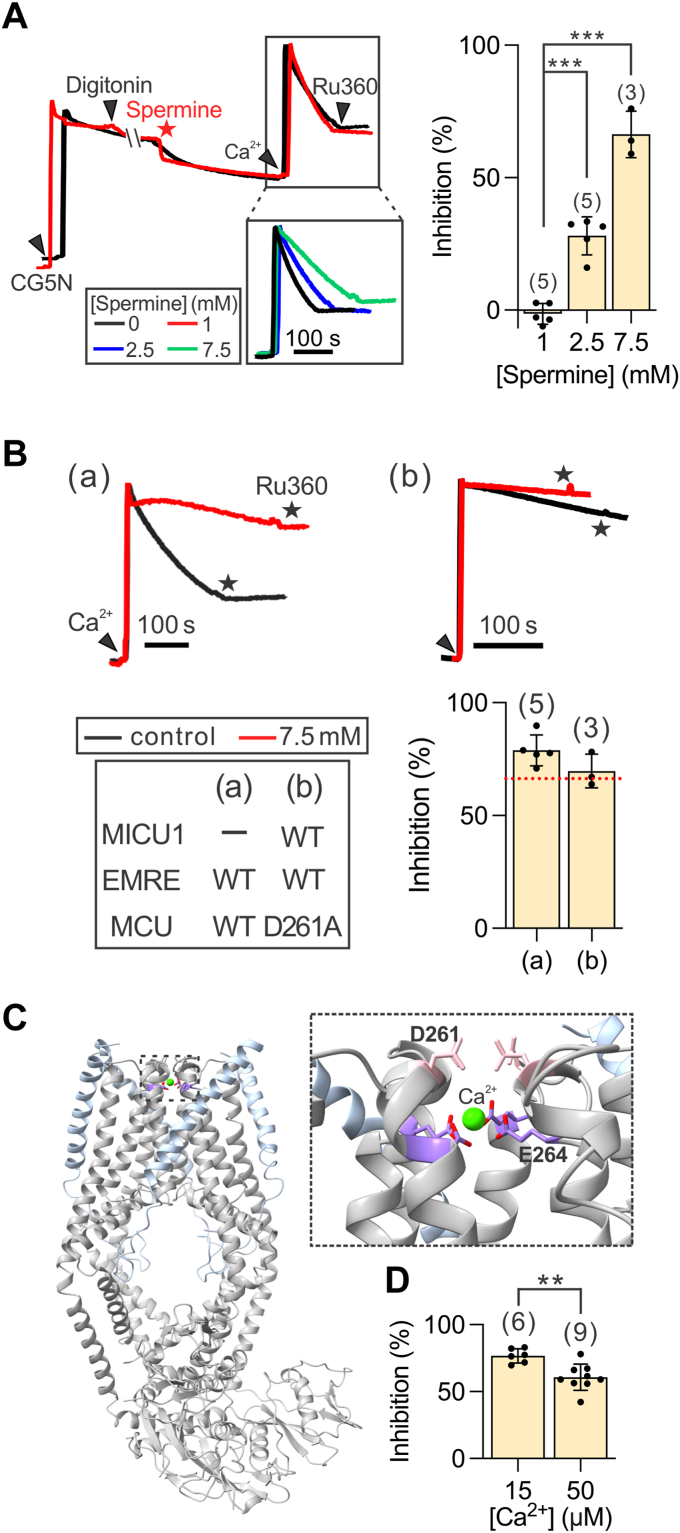
**Dose dependence and mechanistic insights into spermine-mediated inhibition of the uniporter.***A*, spermine inhibition of mitochondrial Ca^2+^ uptake. The bar chart presents the percentage inhibition at various concentrations of spermine. *B*, the effect of MICU1 KO and the MCU D261A mutation on spermine inhibition. *Dashed line*: the level of spermine inhibition in WT uniporter as shown in panel *A*. *Arrowheads* represent 15 μM Ca^2^⁺, while *stars* denote the presence of 75 nM Ru360. *C*, structure of the MCU–EMRE complex (PDB: 6O58). The *dashed box* highlights the two Ca^2+^-binding sites at the cytoplasmic entrance of the pore, formed by the D261 residue and the E264 residue, which are separated by one helical turn. The MCU subunit is shown in *gray*, and EMRE is depicted in *light blue*. *D*, modulation of spermine inhibition by [Ca^2+^]. Statistical analysis was performed using a two-tailed, unpaired *t* test. ∗∗*p* < 0.01; ∗∗∗*p* < 0.001. In the bar charts, each dot represents an independent biological replicate. KO, knockout; WT, wildtype.

We next investigated whether spermine inhibits the uniporter, given its well-established role in blocking cation channels (25) and previous evidence suggesting that it might suppress mitochondrial Ca^2+^ uptake under certain conditions (36, 37, 38, 41, 42, 45). To this end, permeabilized WT HEK cells were pretreated with 1 to 7.5 mM spermine, and after [Ca^2+^]_ex_ dropped to a steady state, 15 μM Ca^2+^ was added to fully release MICU1 block (Fig. 6*A*). Without MICU1 block, spermine does not potentiate the uniporter (Fig. 2, *D* and *E*). Therefore, comparing the initial rates of mitochondrial Ca^2+^ uptake immediately after adding 15 μM Ca^2+^ with or without spermine could inform us whether spermine inhibits the uniporter (box, Fig. 6*A*). No inhibition was detected with 1 mM spermine, but the Ca^2+^ uptake rate was reduced by ∼25% and ∼70% at 2.5 and 7.5 mM spermine, respectively (Fig. 6*A*). These results demonstrate that spermine inhibits the uniporter, albeit with a potency more than 10-fold lower than its stimulatory effect.

Further experiments show that 7.5 mM spermine reduces the rate of mitochondrial Ca^2+^ uptake in MICU1-KO cells similarly to WT cells (Fig. 6, *A* and *B*). Since MICU1-KO cells lack MICU dimers in the uniporter complex, this result indicates that, unlike its potentiation effect, spermine inhibition of the uniporter does not depend on MICU1. The fact that spermine is a well-known pore blocker of various cation channels (25) led us to hypothesize that it may interact with the Ca^2+^-binding region inside the MCU pore (Fig. 6*C*). Supporting this hypothesis, increasing [Ca^2+^] alleviates uniporter inhibition by 7.5 mM spermine in both WT cells (Fig. 6*D*), suggesting that spermine competes with Ca^2+^ for the Ca^2+^-binding site.

MCU possesses two Ca^2+^-binding sites: a loose site and a tight site, formed by the tetrameric side-chain carboxyl rings of D261 and E264 (55, 56, 57, 58), respectively (Fig. 6*C*). The D261A mutation does not affect inhibition of the uniporter by 7.5 mM spermine (Fig. 6*B*), indicating that D261 is not essential for spermine’s inhibitory effect. Unfortunately, we cannot experimentally assess the role of E264, as mutations of E264 abolish uniporter function (13).

## Discussion

**Figure 7:**
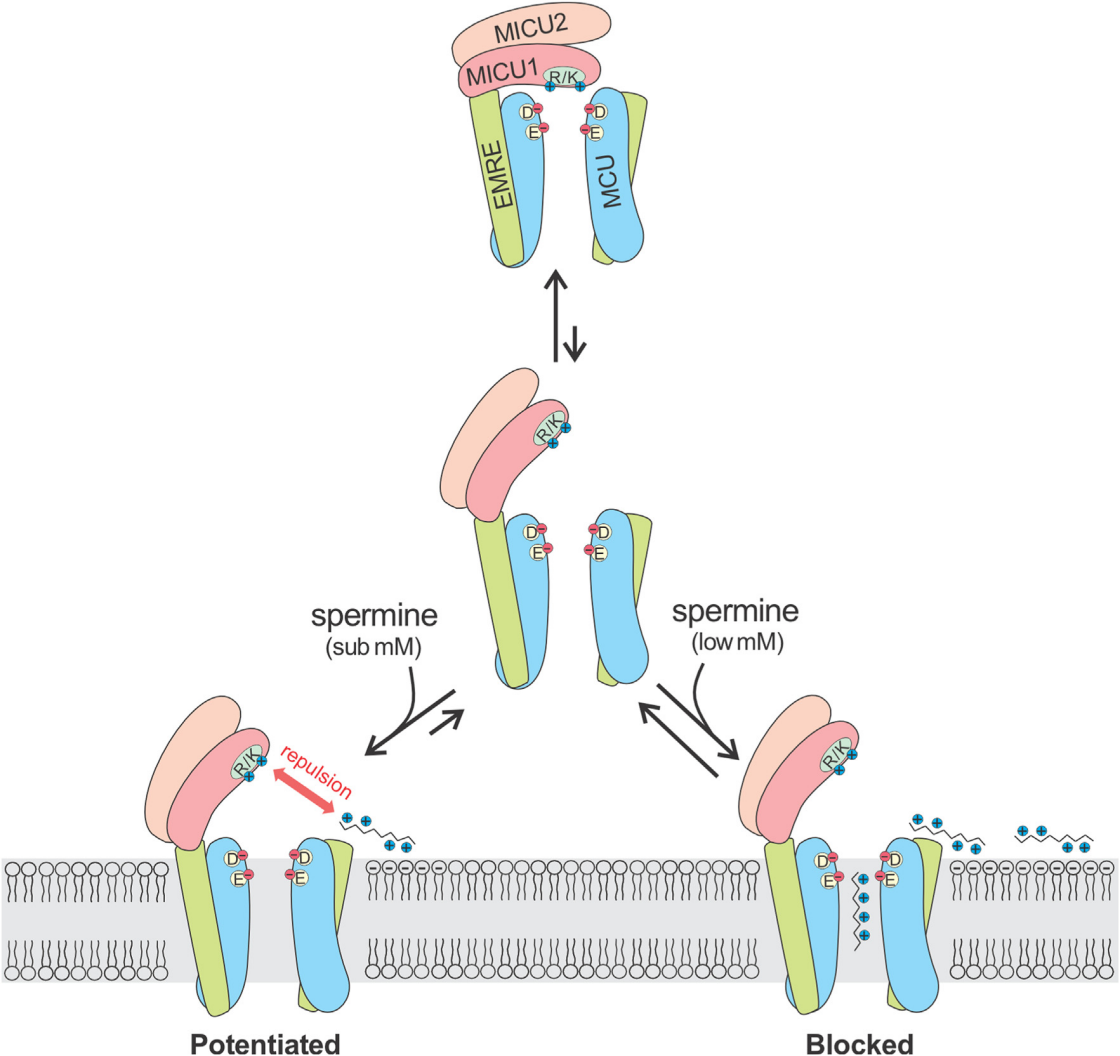
**Proposed mechanisms of spermine’s potentiation and inhibition of the uniporter.** The cartoon illustrates that at resting cellular Ca^2^⁺ levels, the uniporter predominantly exists in an occluded state (*top*), where the MICU1 subunit closes the uniporter by using Arg and Lys residues to interact with MCU’s Asp residue at the pore entrance. At sub-millimolar spermine levels (*bottom left*), the positive charge of membrane-bound spermine molecules creates electrical repulsion with MICU1, preventing MICU1 from closing the pore. However, at millimolar spermine levels (*bottom right*), spermine enters the pore, leading to blocking. This proposed mechanism reconciles the dual effects of spermine reported in the literature.

This work demonstrates that spermine potentiates the uniporter by disrupting MICU1-mediated occlusion of the channel’s pore (Fig. 7). Notably, decades-old observations have already hinted at such a mechanism. It has been well-established that the rate of mitochondrial Ca^2+^ uptake rises along a sigmoidal curve with increasing [Ca^2+^], because increased [Ca^2+^] cooperatively relieves MICU1 block of MCU to open the uniporter. Kröner reported that spermine altered this Ca^2+^ dose–response curve from sigmoidal to linear (41), indicating that spermine impairs MICU1 regulation over the uniporter (9, 16, 19). This observation thus supports our proposed mechanism that spermine disrupts MICU1 block.

The findings that spermine, aminoglycoside antibiotics, and basic peptides (*e.g.*, poly-L-lysine)—despite having low structural similarity other than their positive charge—can all enhance uniporter activity, and that these molecules all bind well to lipid bilayers, have long led to the speculation that spermine interacts with the negatively charged phospholipid head groups at the membrane surface to exert its stimulatory effects (37). This idea is now supported by our binding assay, which demonstrates that lipids are crucial for spermine to interrupt MCU-MICU1 interactions.

Based on our findings and recent structural and functional advances revealing that MICU1 occludes the MCU pore through interactions between positively charged Arg and Lys residues in MICU1 and the negatively charged Asp ring at the MCU pore entrance (10, 11, 12, 13, 14), we propose a mechanism for spermine’s enhancement of mitochondrial Ca^2+^ sequestration (Fig. 7). As mitochondria absorb and reduce external Ca^2+^, the uniporter enters into a MICU1-occluded, inactive state that ceases to import Ca^2+^. In the presence of spermine, when MICU1 occasionally dissociates from MCU, the membrane-bound spermine would generate an electrical repulsion with MICU1. This prevents MICU1 from re-blocking MCU, shifting the uniporter’s gating equilibrium toward a MICU1-unblocked, conducting state and thereby activating the uniporter to allow mitochondria to sequester more Ca^2+^.

Our results provide an explanation for the puzzling observation that spermine stimulates mitochondrial Ca^2+^ uptake in hepatic mitochondria but not in cardiac mitochondria (41) (but see another reference (43)). This phenomenon can now be understood by integrating our current findings with recent research (9), which shows that cardiac mitochondria have very low levels of MICU1, leading to a large population of MICU1-free uniporters that exhibit a linear [Ca^2+^] dose response (9, 59). According to our proposed spermine potentiation mechanism (Fig. 7), these MICU1-deregulated cardiac uniporters would not become more active in response to spermine, aligning with the observed lack of stimulation reported in the literature.

While this manuscript was in preparation (60), another group published a report investigating the mechanisms of uniporter activators (61). They proposed that activators such as spermine and kaempferol enhance uniporter activity through a general MICU1-dependent mechanism, likely by binding near MICU1’s EF hands and suppressing its gatekeeping function. It was further suggested that spermine might synergize with Ca^2+^ to induce conformational changes in MICU1, thereby relieving its occlusion of the MCU pore (61). In contrast, our findings support a fundamentally different mechanism: spermine interacts with lipids, rather than directly engaging with MICU1, and prevents MICU1-mediated block of the uniporter through electrical repulsion (Fig. 7). Furthermore, we demonstrated that kaempferol does not act as an activator of mitochondrial Ca^2^⁺ uptake.

We found that spermine can also inhibit the uniporter and propose that this inhibition occurs through spermine blocking the MCU pore (Fig. 7) for the following reasons. First, spermine inhibition only requires the uniporter’s pore-forming subunits, independent of the regulatory MICU1 and MICU2 subunits. Second, spermine inhibits other cation channels all *via* pore-blocking mechanisms, consistent with its linear molecular structure, which is well-suited for entering and obstructing channel pores. Finally, the observation that spermine competes with Ca^2+^ for binding to MCU indicates that spermine and Ca^2+^ share the same site in MCU. The physiological relevance of spermine’s inhibitory effect remains to be explored. While the IC_50_ is in the millimolar range—significantly higher than typical intracellular spermine concentrations—it is possible that spermine could be concentrated at bilayer surfaces to levels sufficient to inhibit the uniporter.

Our investigation sheds light on the complex and seemingly contradictory effects of spermine on mitochondrial Ca^2+^ uptake kinetics. In particular, multiple labs (36, 41, 42) have reported that spermine's effects shift from stimulatory to inhibitory as [Ca^2+^] increases. This transition likely occurs because spermine's stimulatory effect is more pronounced at low [Ca^2+^], where MICU1 blocks MCU, while the inhibitory effect emerges when Ca^2+^ elevation relieves MICU1 block and diminishes spermine’s ability to potentiate. Additionally, some researchers have observed only spermine potentiation (40) or inhibition (37, 38, 45). This variability could be attributed to differences in spermine concentrations, which might fall within the ranges where either the stimulatory or inhibitory effects dominate.

In summary, this study elucidates the mechanisms by which spermine regulates uniporter function. These findings pave the way for future work to understand how spermine could act as a signaling molecule to modulate crucial Ca^2+^-dependent mitochondrial processes in mammalian cells, both under physiological conditions and pathological states.

## Experimental procedures

### Cell culture and molecular biology

Human embryonic kidney 293 cells were obtained from ATCC (#CRL-11268), authenticated using short tandem repeat profiling, and cultured in Dulbecco's Modified Eagle Medium supplemented with 10% fetal bovine serum at 37 °C in a 5% CO_2_ incubator. CRISPR/Cas9-mediated deletion of uniporter-subunit genes in HEK cells was performed as described in our previous work (15, 62). For transient expression, codon-optimized uniporter genes were cloned into the pcDNA3.1 vector and transfected into cells using Lipofectamine 3000 (Thermo Fisher) according to the manufacturer's instructions. Transfected HEK cells were harvested for all experiments in this work 48 h posttransfection. Site-directed mutagenesis was carried out using the QuickChange kit (Agilent), and all constructs were verified by Sanger sequencing.

### Mitochondrial Ca^2+^ flux assay

HEK cells (2 × 10^7^) were washed in 10 ml of wash buffer (120 mM KCl, 25 mM Hepes, 2 mM KH_2_PO_4_, 1 mM MgCl_2_, 50 μM EGTA, and pH 7.2-KOH) and subsequently resuspended in 2 ml of recording buffer (120 mM KCl, 25 mM Hepes, 2 mM KH_2_PO_4_, 1 mM MgCl_2_, 5 mM succinate, and pH 7.2-KOH). The sample was then transferred into a stirred quartz cuvette and placed in a Hitachi F-7100 spectrofluorometer for fluorescence measurements with temperature maintained at 25 °C. The spectrofluorometer parameters were set as follows: for Fluo-4 (Thermo Fisher, F14200), excitation at 494 nm and emission at 516 nm; for Calcium Green 5N (CG5N, Thermo Fisher, C3737), excitation at 508 nm and emission at 531 nm. For both dyes, the excitation slit width was 2.5 nm, the emission slit width was 5.0 nm, and the sampling frequency was 1 Hz. Fluorescence readings are expressed in arbitrary units (A.U.).

To analyze spermine’s potentiation effects on mitochondrial Ca^2+^ buffering, 0.25 μM Fluo-4 was added to the cell suspension to monitor [Ca^2+^]_ex_, followed by 30 μM digitonin (Sigma, D141) to permeabilize the cells. After [Ca^2+^]_ex_ was buffered to a steady state, spermine was applied to induce further Ca^2+^ sequestration into mitochondria. After the fluorescence signal stabilized, 40 μM CaCl_2_ was added to obtain the saturating fluorescence (F_max_), followed by 500 μM EGTA to obtain the minimum fluorescence signal (F_min_). [Ca^2+^]_ex_ was calculated using the equation:

$${\left[{Ca}^{2+}\right]}_{ex}=K_{d}\cdot \frac{\left(F-F_{\min}\right)}{\left(F_{\max}-F\right)}$$

where F represents the average fluorescence signal over a 10-s window, centered on the time point of interest, and the dissociation constant (K_d_) value for Fluo-4 is 345 nM, as specified by the manufacturer. The percentage decrease in [Ca^2+^]_ex_ (D) caused by spermine was calculated as follows:

$$D=\frac{{\left[Ca^{2+}\right]}_{0}-{\left[Ca^{2+}\right]}_{S}}{{\left[Ca^{2+}\right]}_{0}}$$

where [Ca^2+^]_0_ and [Ca^2+^]_s_ represent the steady-state [Ca^2+^]_ex_ before and after applying spermine, respectively.

To assess the effect of spermine on the rate of mitochondrial calcium uptake (*e.g.*, Figs. 2*E* and 6*A*), CG5N was used to monitor [Ca^2+^]_ex_. Reagents were added in the following sequence: 0.5 μM CG5N, 30 μM digitonin, varying concentrations of spermine, varying concentrations of CaCl_2_, 75 nM Ru360, 1 mM CaCl_2_ to determine F_max_, and finally 2 mM EGTA to establish F_min_. The rate of calcium uptake (R) was calculated using the equation:

$$R=\frac{\Delta {\left[Ca^{2+}\right]}_{ex}}{20}$$

where Δ[Ca^2+^]_ex_ represents the reduction of [Ca^2+^]_ex_ during the first 20 s after initiating mitochondrial Ca^2+^ uptake with the addition of Ca^2+^. In Fig. 2*E*, the fold increase represents the ratio of the mitochondrial Ca^2+^ uptake rate with spermine to that without spermine. In Fig. 6, *A* and *B*, spermine inhibition (I) was calculated as follows:

$$I=\frac{R_{NS}-R_{S}}{R_{NS}}$$

where R_S_ and R_NS_ represent the rates of mitochondrial Ca^2+^ uptake with and without spermine, respectively. All mitochondrial Ca^2+^ flux data were analyzed using Igor Pro 8 (WaveMetrics).

### CoIP and Western blots

All CoIP steps were performed at 4 °C unless otherwise specified. For experiments in Figs. 4 and S1-2, transfected HEK cells from a 10-cm dish were suspended in 1 ml of ice-cold solubilization buffer (SB, 150 mM NaCl, 50 mM Hepes, 4 mM DDM, and pH 7.4-NaOH). The SB was supplemented with a protease inhibitor cocktail (Roche, cOmplete EDTA-free) and either 1 mM EGTA, 1 mM spermine, or 10 μM CaCl_2_. After 10 min of incubation, the lysate was clarified by centrifugation at 13,000*g* for 10 min. A 100 μl aliquot of the supernatant was reserved as the whole-cell lysate sample. To the remaining supernatant, 25 μl of either FLAG-antibody-conjugated resin (Sigma Aldrich, A2220) or 1D4-antibody-conjugated resin (homemade, 50% slurry) was added, followed by incubation on a tube revolver for 1 h. The beads were then collected on a spin column, washed six times with 1 ml SB, and proteins were eluted with 140 μl of 1× SDS loading buffer to generate the IP samples.

For experiments in Figs. 5*F* and S3, cell were resuspended in a DDM-free SB and permeabilized with 30 μM digitonin for 2 min at room temperature. The samples were then divided into two aliquots and proceeded under two distinct conditions. In the first condition, permeabilized cells were incubated with 1 mM spermine for 10 min, followed by solubilization with 1 ml SB containing 4 mM DDM for 10 min at room temperature. In the second condition, permeabilized cells were kept without spermine for 10 min, then solubilized with 1 ml SB containing both 4 mM DDM and 1 mM spermine for 10 min at room temperature. After clarification of the lysates by centrifugation (13,000*g*, 10 min, 4 °C), 25 μl of 1D4-antibody-conjugated resin (50% slurry) was added to each sample. The mixtures were then incubated at 4 °C for 1 h on a tube revolver. Subsequent wash and elution steps were performed as described above.

Protein samples were subjected to SDS-PAGE and then transferred to low-fluorescence PVDF membranes. Membranes were blocked in Tris-buffered saline-based Intercept blocking buffer (Li-Cor) and then incubated overnight at 4 °C with primary antibodies diluted in Tris-buffered saline plus 0.075% Tween-20. The following primary antibodies were used: anti-1D4 (TETSQVAPA) (produced in house, 0.1 μg/ml), anti-MICU1 (Sigma Aldrich HPA037480, 1:10,000), anti-actin (Santa Cruz sc-69879, 1:2000), and anti-FLAG (Sigma Aldrich F1804, 1:10,000). (The anti-1D4 antibody was validated by blotting against a transiently expressed protein containing the 1D4 tag, whereas other antibodies were validated against native proteins in cell lysates as specified in the manufacturers’ datasheets.) After washing, membranes were incubated for 1 h at room temperature with fluorescent secondary antibodies: goat anti-rabbit IRDye 680RD (Li-Cor 92568171, 1:10,000) or goat anti-mouse IRDye 680RD (Li-Cor 925-68070, 1:15,000). Fluorescent signals were captured using a LI-COR Odyssey CLx imager, and band intensities were quantified using LI-COR Image Studio software (version 5.2).

### IMM depolarization essay

HEK cells were grown to confluence in 10-cm dishes. The culture medium was replaced with 10 ml Tyrode’s solution (130 mM NaCl, 5.4 mM KCl, 1 mM MgCl_2_, 1 mM CaCl_2_, and 20 mM Hepes, pH 7.8-NaOH) containing 40 nM tetramethylrhodamine, methyl ester (TMRM; Thermo Fisher, T668). After incubating at 37 °C for 30 min, cells were washed with TMRM-free Tyrode’s solution. Subsequently, 2 × 10^7^ cells were suspended in 10 ml of Mg^2+^-free wash buffer (120 mM KCl, 2 mM K_2_HPO_4_, 50 μM EGTA, and 25 mM Hepes, pH 7.2-KOH). Following centrifugation, the cell pellet was resuspended in 2 ml Mg^2+^-free recording buffer (120 mM KCl, 2 mM K_2_HPO_4_, 5 mM succinate, and 25 mM Hepes, pH 7.6-KOH). The sample was transferred to a stirred quartz cuvette and analyzed using a Hitachi F-7100 spectrofluorometer with the following settings: temperature 25 °C, excitation wavelength 573 nm (slit width 5 nm), emission wavelength 590 nm (slit width 5 nm), and sampling frequency 1 Hz. After cells were permeabilized with 30 μM digitonin, 1 mM spermine was added, followed by the addition of 1 μg/ml carbonyl cyanide-4-(trifluoromethoxy)phenylhydrazone to completely collapse the IMM potential. The rate of IMM depolarization was quantified by normalizing the slope before and after spermine addition to the total TMRM signal.

### Tryptophan fluorescence titration

WT MICU1 fused with an N-terminal maltose-binding protein was purified *via* affinity purification and size-exclusion chromatography as described in our previous publication (18). The purified protein was diluted to 0.5 μM in 2 ml of buffer (500 mM NaCl, 25 mM Hepes, 250 μM BAPTA, pH 7.5-NaOH) and transferred into a stirred quartz cuvette. Changes in tryptophan fluorescence in response to sequential additions of 30 μM Ca^2+^ and 0.5 mM spermine were monitored using the Hitachi F-7100 spectrophotometer. Measurements were conducted under the following conditions: temperature 25 °C, excitation wavelength 280 nm (slit width 2.5 nm), emission wavelength 350 nm (slit width 5 nm), and sampling frequency 1 Hz.

### Statistics

Statistical analyses throughout the manuscript were performed using Microsoft Excel with unpaired two-tailed Student’s *t* test. Significance was defined as *p* < 0.05. All experiments were conducted with a minimum of three independent replicates. Data are presented as mean ± standard deviation (SD).

## Data availability

All data supporting this study are included within the manuscript. Uncropped, original Western blot images for Figs. 2*C*, 4, *A* and *B*, and 5*F* are provided in Fig. S4. Independent replicates for the Western blot experiments shown in Figs. 4, *A* and *B* and 5*F* are available in Figs. S1–S3. Raw data used to generate the bar graphs throughout the manuscript can be accessed *via* Mendeley Data (https://doi.org/10.17632/xjhxvmmng9.1).

## Supporting information

This article contains supporting information including Figs. S1–S4.

## Conflict of interest

The authors declare that they have no conflicts of interest with the contents of this article.
