# Supplementary material for: Mechanisms of dual modulatory effects of spermine on the mitochondrial calcium uniporter complex
Source: J Biol Chem. 2025 Jan 23;301(3):108218. doi: 10.1016/j.jbc.2025.108218 (PMC11871460; doi:10.1016/j.jbc.2025.108218)
Supplement: Supporting Information [file mmc1.pdf]

Supporting Information

Mechanisms of dual modulatory effects of spermine on the mitochondrial calcium uniporter complex

Yung-Chi Tu, I-Chi Lee, Tsai-Wei Chang, Vivian Lee, Fan-Yi Chao, Eitel R. Geltser, & Ming-Feng Tsai

This file includes Figures S1 to S4.

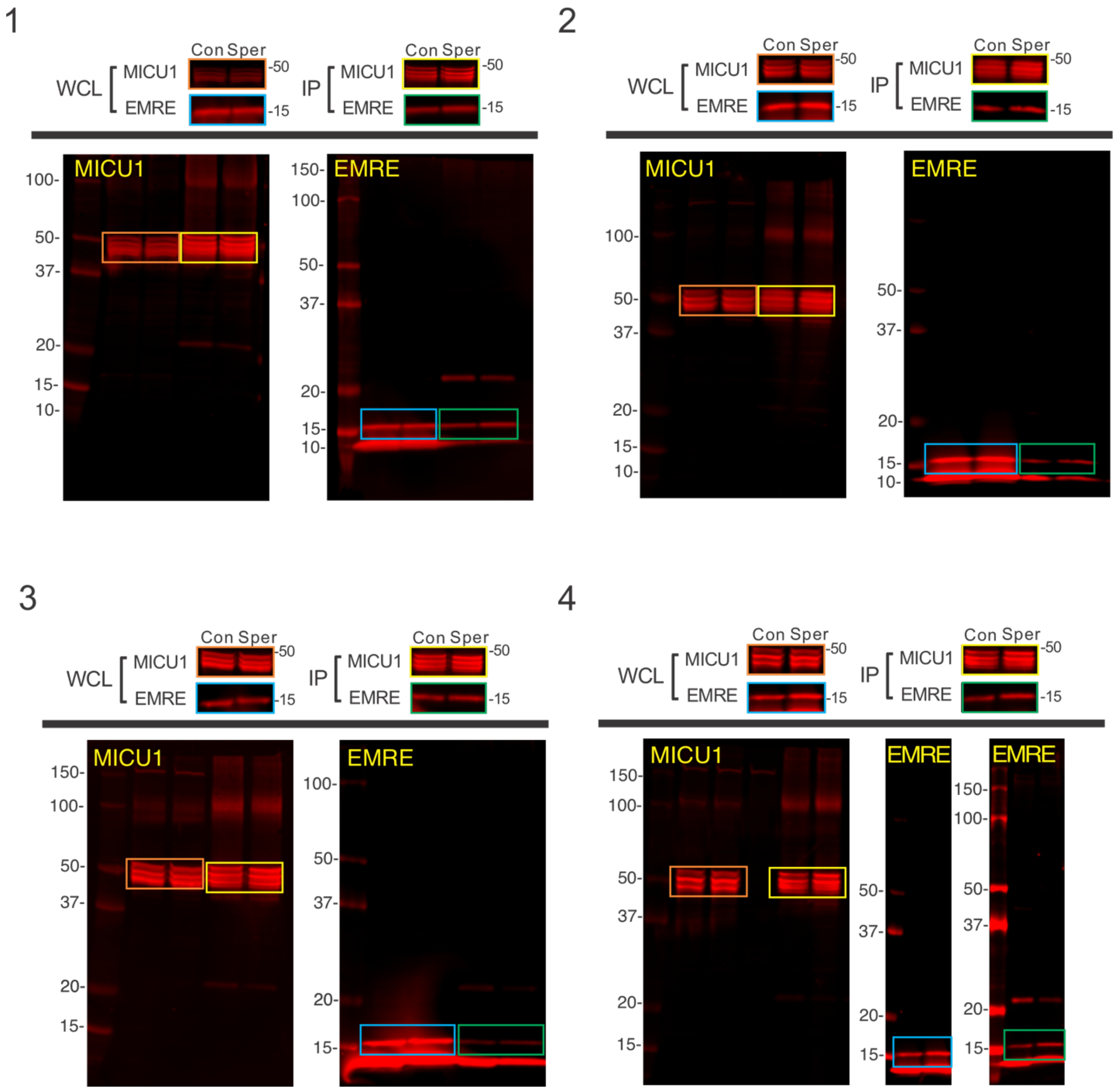

Figure S1. Co-immunoprecipitation (CoIP) analysis of spermine's effects on MICU1-EMRE interactions. This figure supplements Fig. 4A by presenting results from four additional independent biological replicates (panels 1–4). Each panel is divided into two sections: the upper portion shares the same format of Fig. 4A, while the lower portion displays the corresponding uncropped Western blot images. Color-coded boxes link cropped bands to their respective uncropped images. The numbers indicate molecular weight markers in kDa.

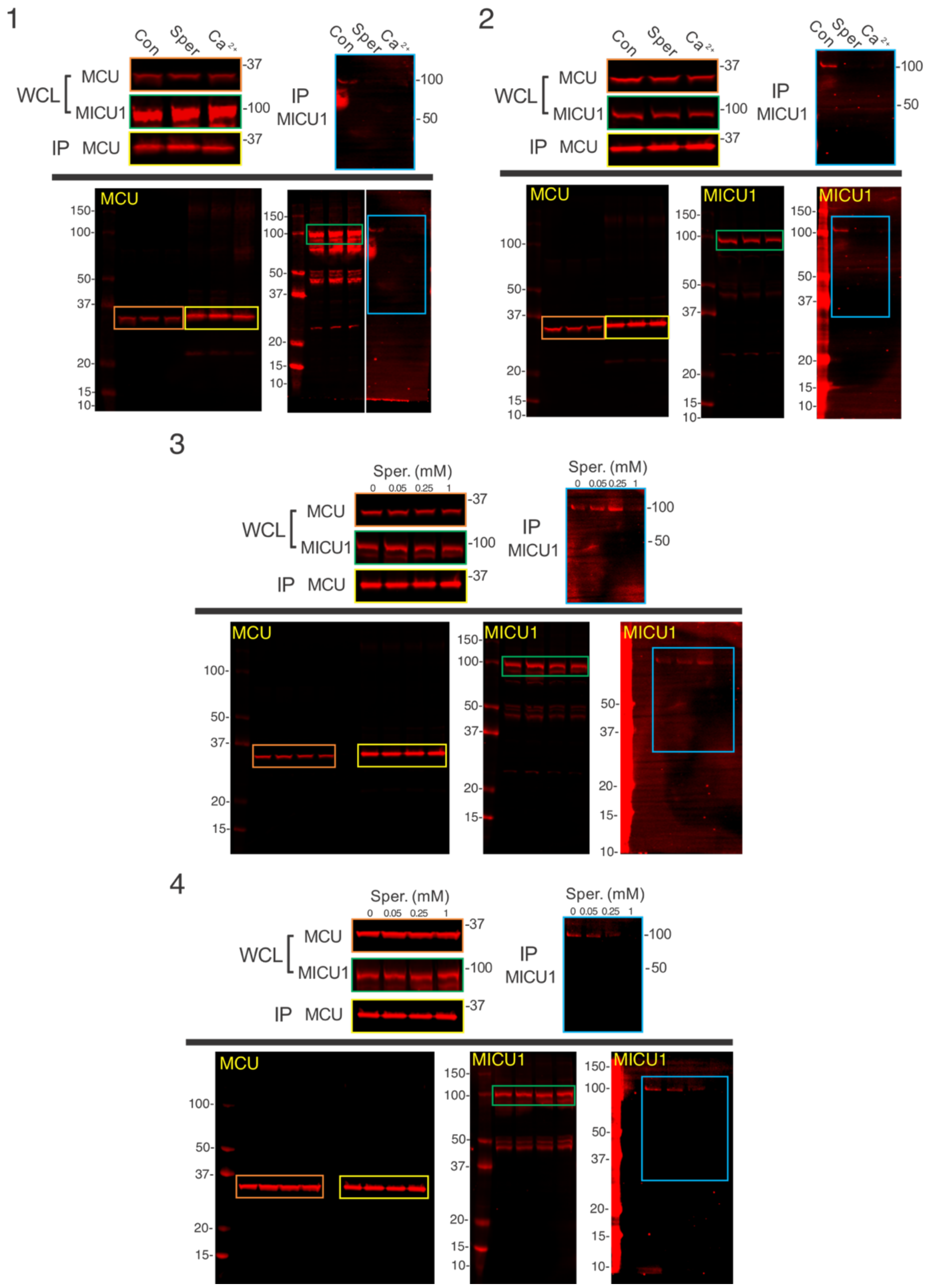

Figure S2. Co-immunoprecipitation analysis of spermine's effects on MICU1-MCU interactions. This figure supplements Fig. 4B by presenting data from four additional independent biological replicates (panels 1–4). Each panel consists of two sections: the upper portion is presented using the same format of Fig. 4B, while the lower portion displays the corresponding uncropped Western blot images. Color-coded boxes connect cropped bands to their respective uncropped counterparts for clarity. Experiments in Fig. 4B and panels 1–2 used 1 mM spermine, while panels 3–4 tested additional spermine concentrations, revealing a dose-dependent disruption of MICU1-MCU interactions.

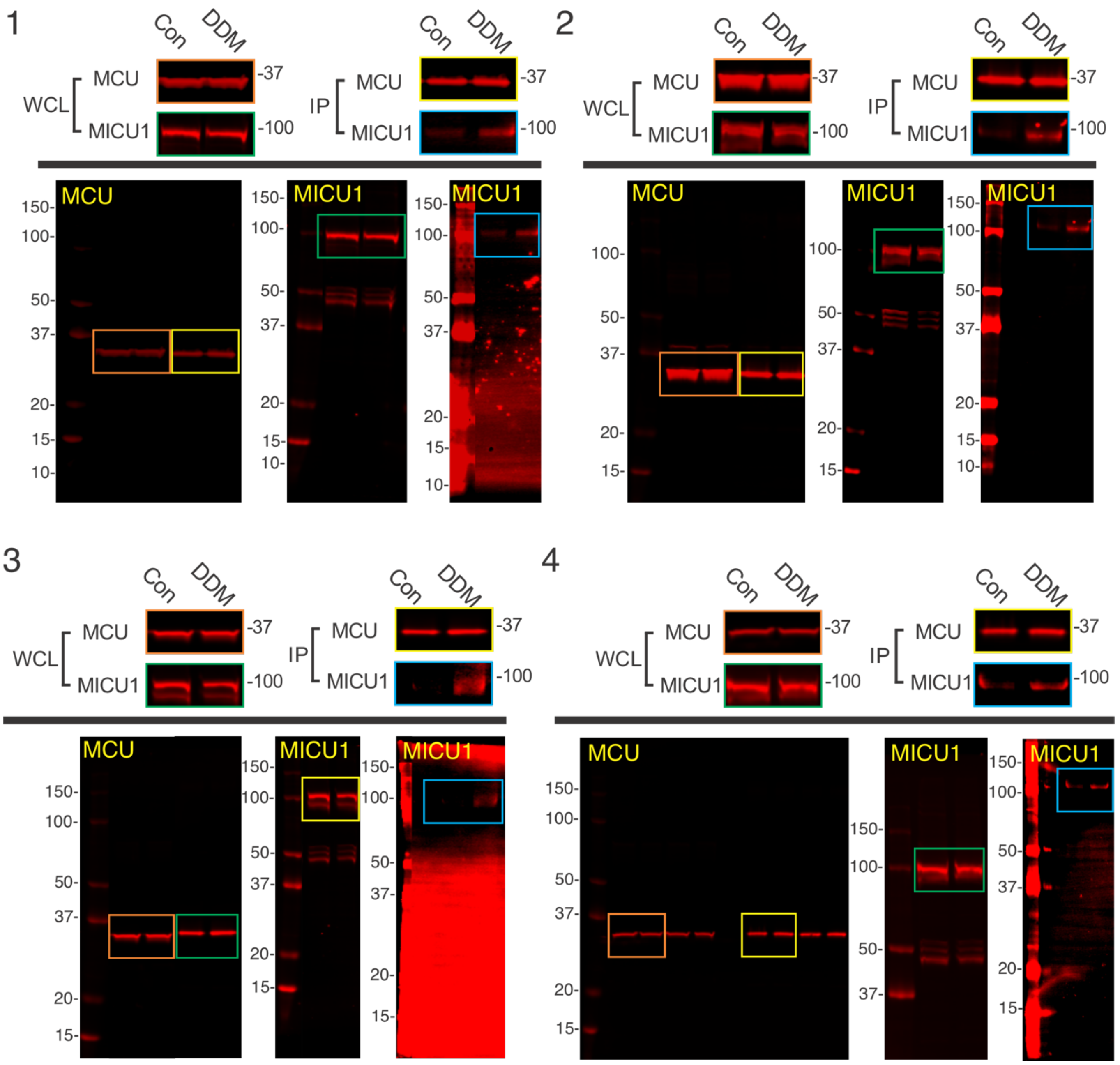

Figure S3. Lipid dependence of spermine potentiation analyzed via CoIP experiments. This figure provides supplementary information for Fig. 5F by presenting data from four additional independent biological replicates (panels 1–4). Each panel consists of two sections: the upper portion is formatted as in Fig. 4B, while the lower portion shows the corresponding uncropped Western blot images. Color-coded boxes link cropped bands to their uncropped counterparts. Experimental conditions are identical to those described in Fig. 5F.

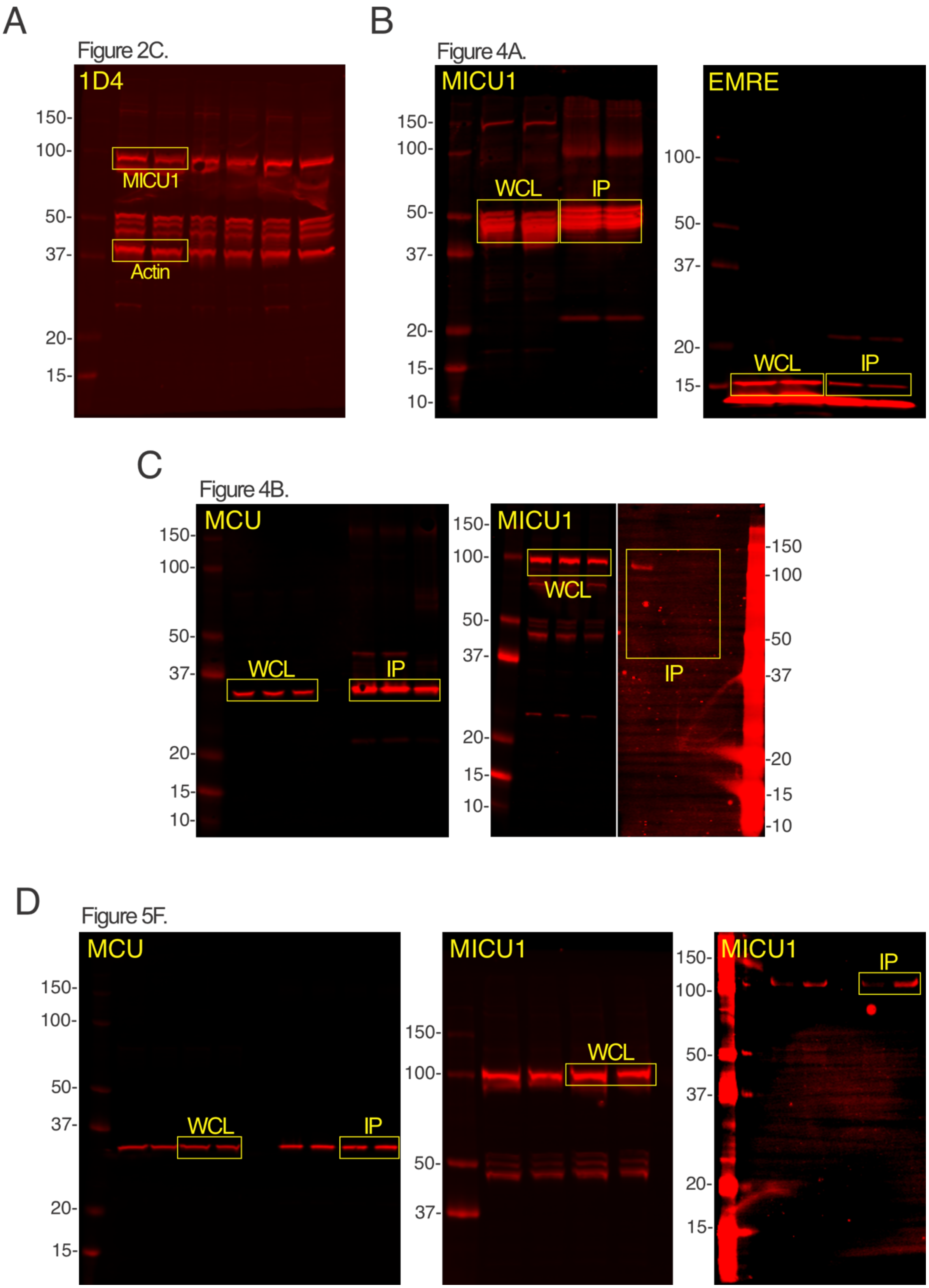

Figure S4. Uncropped Western blot images for main figures. This figure provides the original uncropped Western blot images corresponding to the main figures: Fig. 2C (A), Fig. 4A (B), Fig. 4B (C), and Fig. 5F (D). Yellow boxes highlight the bands displayed in the main figures.
